# Use of a *Drosophila* Genome-Wide Conserved Sequence Database to Identify Functionally Related *cis*-Regulatory Enhancers

**DOI:** 10.1002/dvdy.22728

**Published:** 2011-08-30

**Authors:** Thomas Brody, Amarendra S Yavatkar, Alexander Kuzin, Mukta Kundu, Leonard J Tyson, Jermaine Ross, Tzu-Yang Lin, Chi-Hon Lee, Takeshi Awasaki, Tzumin Lee, Ward F Odenwald

**Affiliations:** 1 Neural Cell-Fate Determinants Section, NINDS, NIHBethesda, Maryland; 2 Division of Intramural Research, Information Technology Program, NIHBethesda, Maryland; 3 Section on Neuronal Connectivity, NICHD, NIHBethesda, Maryland; 4 HHMI, Janelia Farm Research CampusAshburn, Virginia

**Keywords:** *cis*-regulatory enhancers, conserved sequence clusters (CSCs), *Drosophila melanogaster*

## Abstract

Background: Phylogenetic footprinting has revealed that *cis*-regulatory enhancers consist of conserved DNA sequence clusters (CSCs). Currently, there is no systematic approach for enhancer discovery and analysis that takes full-advantage of the sequence information within enhancer CSCs. Results: We have generated a *Drosophila* genome-wide database of conserved DNA consisting of >100,000 CSCs derived from *EvoPrints* spanning over 90% of the genome. *cis*-Decoder database search and alignment algorithms enable the discovery of functionally related enhancers. The program first identifies conserved repeat elements within an input enhancer and then searches the database for CSCs that score highly against the input CSC. Scoring is based on shared repeats as well as uniquely shared matches, and includes measures of the balance of shared elements, a diagnostic that has proven to be useful in predicting *cis*-regulatory function. To demonstrate the utility of these tools, a temporally-restricted CNS neuroblast enhancer was used to identify other functionally related enhancers and analyze their structural organization. Conclusions: *cis*-Decoder reveals that co-regulating enhancers consist of combinations of overlapping shared sequence elements, providing insights into the mode of integration of multiple regulating transcription factors. The database and accompanying algorithms should prove useful in the discovery and analysis of enhancers involved in any developmental process. Developmental Dynamics 241:169–189, 2012. © 2011 Wiley Periodicals, Inc.

**Key findings:**

- A genome-wide catalog of Drosophila conserved DNA sequence clusters.
- *cis*-Decoder discovers functionally related enhancers.
- Functionally related enhancers share balanced sequence element copy numbers.
- Many enhancers function during multiple phases of development.

## INTRODUCTION

Understanding the mechanisms of dynamic gene expression remains a major goal of developmental biology. Previous studies have shown that many of the different spatial-temporal aspects of gene regulation are controlled by multiple, functionally independent *cis*-regulatory modules or enhancers (review by Bulger and Groudine, 2011). These studies have also identified several key characteristics of enhancers including their ability to act at some distance from the genes that they regulate, their positional independence relative to transcription direction of the regulated gene, and their ability to function from within transcribed sequences (reviewed by Davidson, 2001). Functional analysis of in vivo characterized enhancers has also revealed that they typically span 300 to 2,000 bp and contain clusters of DNA-binding sites for sequence-specific DNA-binding transcription factors (reviewed by Alonso et al., 2009). More recent studies indicate that some enhancers are regulated by chromatin DNA modifications and/or alterations in higher-order chromatin structure (reviewed by Suganuma and Workman, 2011).

The availability of genomic sequences from evolutionarily related species allows for the comparison of orthologous DNAs, via phylogenetic footprinting, to identify functionally important conserved sequences within enhancers (reviewed by Visel et al., 2007; King et al., 2007; Meireles-Filho and Stark, 2009; Alonso et al., 2009). The conserved enhancer sequence complexity suggests that they integrate multiple regulatory inputs via different sequence-specific DNA-binding factors (Kuo et al., 1998; Berman et al., 2004, Brody et al., 2007). One of the hallmarks of developmental enhancers is the presence of repeated DNA-binding sites for essential transcription factors (Small et al., 1992; Davidson, 1999, Berman et al., 2002, 2004; Gaul, 2010). For example, multiple conserved DNA-binding sites for Hunchback have been identified within *Drosophila* segmentation enhancers (Papatsenko et al., 2009), multiple bHLH DNA-binding sites are found within neural precursor cell enhancers (Brody et al., 2007; Kuzin et al, 2009), and similarly for Runt-, Ets-, and Smad-responsive enhancers in mammals (Bowers et al., 2010; Babayeva et al., 2010; Nakahiro et al., 2010). Studies have also shown that altering the copy number of transcription factor docking sites by adding or deleting multi-copy sequence motifs can alter enhancer behavior. This suggests that such repeat motifs are not necessarily redundant but each conserved copy may have an integral role in enhancer function (Kuzin et al., 2011). In addition, studies on sequentially arrayed or clustered *Drosophila* enhancers have shown that individual enhancers are flanked by sequences referred to as spacers (Small et al., 1993). Comparative genome analysis of spacer regions, termed here inter-clustal regions (ICRs), reveals that they exhibit a higher level of interspecies sequence length variability than do the less-conserved sequences within enhancer-conserved sequence clusters (CSCs) (Kuzin et al., 2009), thus providing a useful method for delimiting the boundaries of enhancers.

Our previous work has described *EvoPrinter*, a phylogenetic footprinting tool for discovering conserved sequences that are shared among orthologous DNAs (Odenwald et al., 2005; Yavatkar et al., 2008). The output of *EvoPrinter*, an evolutionary gene print or *EvoPrint*, portrays in a single readout the conserved DNA within a species of interest, thus highlighting conservation in a continuous gap-free sequence that facilitates the further comparative analysis of enhancer sub-structural organization as well as the discovery of novel enhancers (see below). We have also developed a set of integrated alignment algorithms, collectively known as *cis*-Decoder, that identify multi-copy and unique elements within CSCs that are shared with other CSCs (Brody et al., 2007, 2008).

To increase our understanding of enhancer sub-structure and to identify families of functionally related enhancers via comparative analysis, we constructed a web-accessible genome-wide database of *Drosophila* CSCs that includes, in addition, CSCs within most in vivo characterized enhancers. Also described are additional *cis*-Decoder search algorithms that facilitate the discovery of database CSCs related to any input enhancer. Once the user inputs an *EvoPrinted* enhancer, *cis*-Decoder algorithms scan the database to detect structurally related CSCs using a three-step protocol: the initial search identifies database CSCs that share conserved multi-copy elements with the input sequence; the program then identifies unique elements shared between the input enhancer and database CSCs; and finally the copy number of shared elements is evaluated to generate ranked similarity scores that relate the input enhancer to the database CSCs. To demonstrate the efficacy of this approach, which makes no assumptions about the function of individual sequence elements, we have utilized an enhancer of *castor* (*cas*), a late temporal neuroblast (NB) determinant (Mellerick et al., 1992; Cui and Doe, 1992; Kambadur et al., 1998), to identify previously uncharacterized late NB enhancers. We also show how *cis*-Decoder searches can identify multiple previously characterized cellular gap enhancers based on their shared sequence motifs and also identify shared overlapping transcription factor–binding sites.

Our comparative analysis of enhancers also reveals that there is no single combination of DNA-binding sites of known regulators or novel conserved sequence elements that can accurately predict enhancer regulatory behavior. However, enhancers that have a balance in copy number of shared sequence elements are more likely to exhibit similar regulatory activities. Although enhancers with similar regulatory behaviors share both multi-copy sequence motifs and unique conserved sequence elements that are balanced in copy number, arrangement of these shared elements differs between enhancers. Our studies also demonstrate that many enhancers are multifunctional; they regulate gene expression during different temporal phases of development. No other comparative alignment program allows for the user to generate an inventory of conserved repeat and unique sequences that are shared between CSCs, an essential step in the analysis of their structure. Since the database includes most of the genomic repertoire of CSCs, these tools should serve to help in the further analysis of other novel functionally important sequences and in the discovery of enhancers that drive gene expression during any developmental process or biological event. To our knowledge, this is the first systematic catalog of conserved DNA sequences within any phylogenetic group.

## RESULTS AND DISCUSSION

### Generation of a Genome-Wide CSC Database

**Fig. 1:**
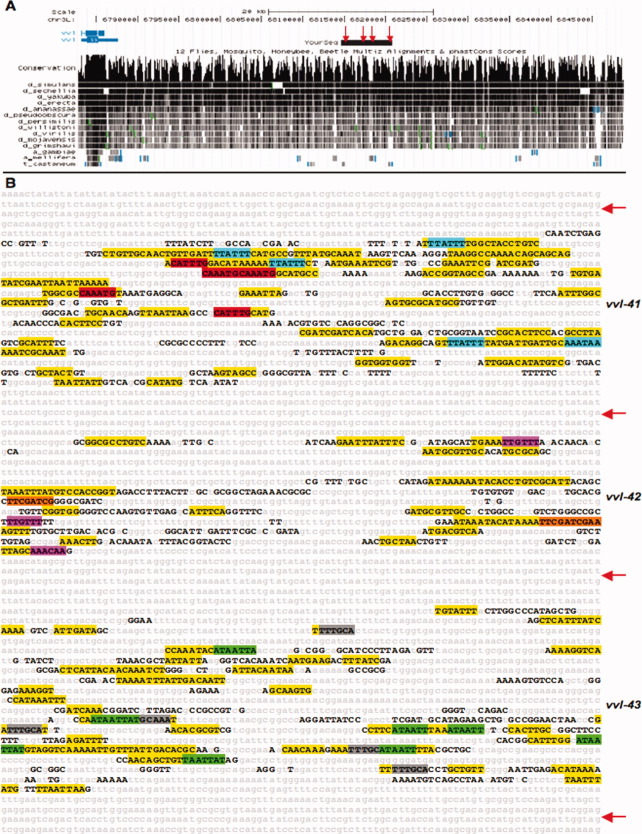
The *Drosophila* genome can be parsed into clusters of conserved sequence blocks that are flanked by less conserved DNA. **A**: Shown is a UCSC Genome Browser conservation histogram of a *D. melanogaster* chromosome 3L region that spans 66 kb of the *vvl* transcribed sequence and 3′ flanking DNA. Highly conserved DNA sequences that align with the orthologous regions of other Drosophilids are indicated as peaks in the histogram. The rectangle identified as “Your Seq” corresponds to the *EvoPrinted* region shown in B and the 4 vertical red-colored arrows correspond to the CSC parsing boundaries shown in B. **B**: A *D. melanogaster* (ref. sequence) relaxed 12 species *EvoPrint* of the Your Seq region in A (6,355 bp) identifies three conserved sequence clusters designated *vvl-41, -42*, and -*43*. Capital letters represent conserved bases in the *D. melanogaster* sequence that are present in all, or in all but one, of the orthologous regions within 11 additional species. Intra-cluster *cis*-Decoder CSB alignments reveal that over 60% of the conserved sequences within each CSC spans ≥ 6-bp repeat sequence elements (yellow highlight) that are either separate, adjacent, and/or overlapping each other. High copy number RPS elements within each CSC are noted with different colored highlights. Red-colored arrows indicate parsing boundaries for the *cis*-Decoder CSC database.

DNA sequence conservation histograms of the *Drosophila* genome reveal that its non-coding DNA is made up of CSCs that are flanked by less-conserved ICR DNA (Karolchik et al., 2007). For example, a conservation histogram of the *Drosophila melanogaster vvl* gene transcribed region and 60 kb of 3′ flanking DNA (located on the 3L chromosome) identifies multiple peaks of conserved DNA that are flanked by less conserved DNA sequences (Fig. 1A). *EvoPrint* analysis reveals that the CSCs can be further resolved into multiple smaller conserved sequence blocks (CSBs) (Fig. 1B). Most regions of chromosomes 2 and 3 gave a similar pattern of CSC density and distribution, while in general CSCs on the X and the 4th chromosomes exhibited less conservation among the twelve species. *cis*-Decoder alignment of CSBs constituting a CSC identifies both repeat and palindromic sequence (RPS) elements, of ≥ 6 bp in length, and reveals that these account for more than half of the CSC's conserved sequences (Fig. 1B). The 6.4-kb genomic region shown in Figure 1B was selected because two of its CSCs (*vvl-41* and *vvl-43*) were tested for their regulatory behavior in this study (see below). Our previous analysis of enhancer sequence conservation has shown that individual enhancers can be identified by the maintenance of their CSB cluster integrity across *Drosophila* species, while ICR regions show greater sequence length variability (Kuzin et al., 2009).

As a first step in the identification of structurally related CSCs, a genome-wide database of *Drosophila* CSCs was created by *EvoPrinting* most of the euchromatic genome of *Drosophila melanogaster* and nearly all of the previously in vivo characterized enhancers that are included in the *REDfly* database (Gallo et al., 2006). Database CSCs were extracted from more than 4,000 author-generated *EvoPrints* that generally spanned 15–30 kb of genomic DNA. *EvoPrints* of fewer bases were used depending on genomic context and availability of gap-free sequence data in the orthologous regions of the different species. Most *EvoPrints* included all of the available *melanogaster* group *drosophilids* (*D. melanogaster, D. simulins, D. sechellia, D. yakuba, D. erecta*, and *D. ananassae*), one of the *obscura* group (*D. pseudoobscura* or *D. persimilis*), and two to four orthologous regions selected from the more evolutionary distant species: *D. willistoni, D. virilis, D. mojavensis*, and/or *D.grimshawi* species. Most of the *EvoPrints* represented a combined evolutionary divergence of >150 My (Tamura et al., 2004). Under these conditions, open reading frames that encode conserved protein domains do not show conservation in most of the codon wobble positions, indicating that the additive evolutionary divergence represented in each *EvoPrint* is sufficient to reveal with near base-pair resolution those sequences that are essential for gene function (Odenwald et al., 2005). *EvoPrints* of open reading frames, using different combinations of species, reveal that the lack of sequence conservation in the amino acid codon wobble position is not the result of different codon preferences between species (data not shown).

To enhance the detection of conserved DNA and avoid alignment inaccuracies triggered by DNA sequencing errors, sequencing gaps, rearrangements, or genome assembly problems that were unique to any one of the species used in the analysis, we employed relaxed *EvoPrint* readouts to identify CSCs. A relaxed *EvoPrint* highlights sequences that are present in all or all but one of the orthologous DNAs used to generate the print (Yavatkar et al., 2008). Species with sequencing gaps (identified as blocks of species-specific differences in the color-coded relaxed *EvoPrint* readouts or identified as gaps in the *EvoPrinter* scorecard) were avoided in generating *EvoPrints*, and second and third scoring pair-wise alignments were included in the analysis when rearrangements were detected (Yavatkar et al., 2008).

**Fig. 2:**
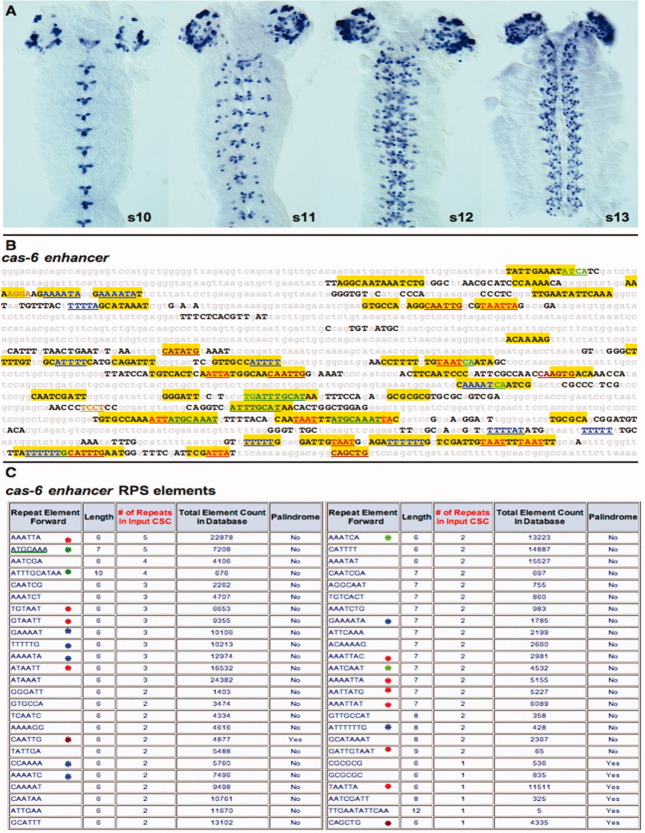
The *cas-6* CSC functions as an NB enhancer that regulates gene expression during late embryonic CNS sub-lineage development. **A**: *cas-6* CSC enhancer-reporter transgene activates expression in a subset of NBs during late sub-lineage development. Shown are dissected fillets of whole-mount stained embryos, stages 10 through 13 (s10–s13; anterior up). **B**: An *EvoPrint* of the *cas-6* enhancer (same *EvoPrint* conditions as in Fig. 1B). CSB sequences that span repeat elements are highlighted in yellow (identified from *cis*-Decoder CSB alignments, see C). Colored underlined bases correspond to the core transcription factor DNA-binding sites (homeodomain, ATTA-red; POU domain, ATGCAAAT-green; bHLH, CANNTG-brown: Hunchback/Castor, TTTTT/AT-blue; Tramtrack, TCCT-gold; and PBX sites, TGAT-teal). **C**: *cis*-Decoder self-alignment of the *cas-6* enhancer CSC identified 50 distinct repeat or palindromic elements. The total element count in the table refers to the number of times a repeat appears in the CSC database. Colored asterisks indicate repeats that contain core known transcription factor DNA-binding motifs highlighted in B. The green-colored underlined repeat indicates the sequence (ATGCAAA) that was used to identify other late sub-lineage NB enhancers that share sequence elements with *cas-6* (see Figs. 3–5).

To catalogue CSCs, *EvoPrints* were entered into the *EvoPrint* CSC cutter algorithm to isolate and annotate individual CSCs separated by at least 150 bp of less-conserved DNA. This program also assigns a file name and consecutive numbers to each CSC in an *EvoPrint*. In order to insure that enhancers that contain CSB separation gaps of 150 bases or more were not truncated, CSCs were also parsed independently two additional times using ICR cutoffs of 200 and 250 bp. Duplicates are given the same name but an additional notation to distinguish them. Therefore, clusters that were parsed multiple times (∼20% of the database CSCs), due to their having non-conserved intervals >150 or >200 but <250 bases, are present two or three times in the database. The database contains >100,000 non-redundant clusters. To expedite database searches, in addition to cataloging individual CSCs and their CSBs, RPS elements of 6 bp or longer were pre-identified by intra-CSC CSB alignments and stored in the database. Most CSCs that contain more than 150 bp of conserved DNA have RPS elements that account for >50 % of their sequences (for example see Fig. 1B; see also Fig. 2B).

The CSC database contains two types of file entries: (1) ∼2,000 files originated from genomic regions spanning previously characterized genes and (2) ∼1,000 entries consisted of genomic regions that cover more than one known or predicted gene or large regions of CSCs not associated with flanking genes. Genomic regions that contain highly repetitive DNA sequences that lack identifiable sequence conservation, such as most of chromosome 4 and specific regions of the X, were not included in the database. Care was taken when annotating the clusters to identify CSCs within non-coding, coding, and 3′UTR regions. Database searches can be modified to include all CSCs or focus on just coding or non-coding regions. It is important to note that CSCs were named according to their proximity to genes: whether the CSCs are indeed enhancers for nearby genes requires functional tests and knowledge of endogenous gene expression patterns. To allow the user to find the location of the CSC relative to flanking genes, a link is provided to the UCSC BLAT server (located on the one-on-one alignment results web-page; see the online *cis*-Decoder tutorial). The UCSC browser also provides information concerning chromatin accessibility and transcription factor–binding data.

We have also included CSCs from all previously in vivo characterized enhancers by *EvoPrinting* all entries in the *REDfly* database (Gallo et al., 2006); these are identified in the CSC-database by their *REDfly* designations. Although most of these CSCs duplicate database entries, CSCs that represent the same region can be identified by their similar *cis*-Decoder scores (see below) and/or their similar identifying names. It should be noted that many *REDfly* entries were made from data that often did not delimit the exact boundaries of the enhancer. In addition many *REDfly* entries included multiple CSCs or truncated CSCs whose ends were restriction enzyme sites used for cloning purposes and were not within less-conserved ICRs. To reduce the number of truncated entries, *EvoPrinted* regions were expanded to include flanking ICRs. Also, since many *REDfly* entries are redundant, care was taken to eliminate this redundancy by eliminating repeated and overlapping entries.

### Identifying Enhancers With Similar Regulatory Behaviors

In addition to the comparative analysis of enhancer sub-structure, our goal in establishing the CSC database and accompanying search algorithms was to identify functionally related enhancers. The assumption that initiated this study is that many functionally related enhancers share overlapping sets of conserved sequence elements. To demonstrate the utility of *cis*-Decoder search algorithms in identifying related tissue- and/or temporal-specific enhancers, we show how a single enhancer can be used to identify other functionally related enhancers. A detailed step-by-step tutorial describing the use of the search protocol is given at the *cis*-Decoder website (http://cisdecoder.ninds.nih.gov/pages/tutorial/index.html).

### CSC Database Search Protocol

The first step in a CSC database search is to enter into the *cis*-Decoder input window an *EvoPrinted* enhancer that spans a single CSC. *cis*-Decoder then parses and annotates constituent CSBs in forward and reverse/complement directions. By alignment of the CSBs to one another, the program next identifies multi-copy and palindromic elements that are ≥6 bp. A table is generated that shows the copy-number of each repeat, the element frequency in the database, and the number of database CSCs that contain two or more of each element. Based on our earlier analysis of known enhancers, matches of less than 6 bp in length were not considered, because searches with 5 bases or less yielded results that were not informative (Brody et al., 2007, 2008; and data not shown).

**Fig. 3:**
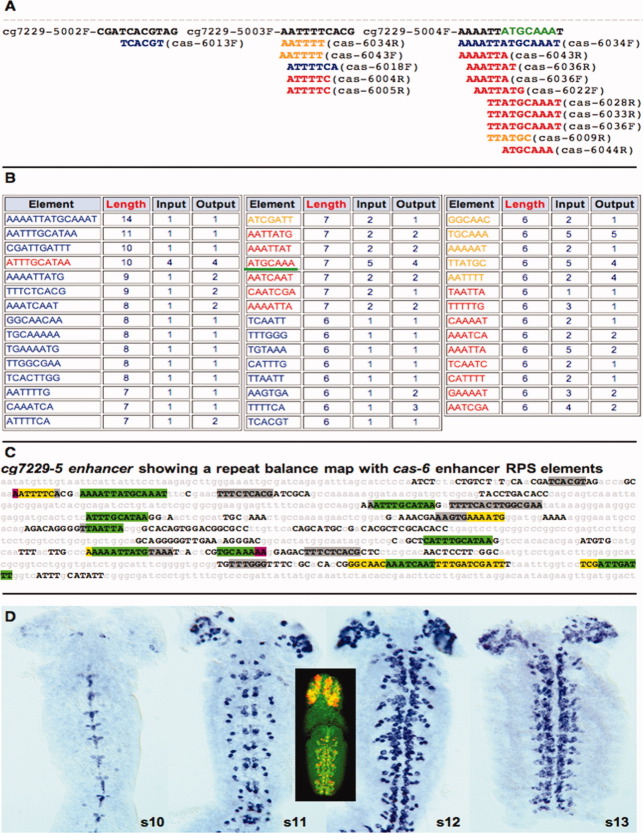
Late sub-lineage NB enhancers share conserved repeat elements that are balanced in their frequency of occurrence. **A**: A one-on-one CSB *cis*-Decoder alignment of three consecutive *cg7229-5* CSBs (nos. 2–4) with *cas-6* CSB sequences. Color-coded bases: Green, the required *cas-6* repeat element used to identify other CSCs in the database search; Blue, sequences are present just once in the *cas-6* enhancer; Red, *cas-6* repeats; Orange, shorter (≥ 6 bp) repeat sequences that are part of larger *cas-6* repeats. The *cas-6* CSB number and alignment orientation (forward or reverse) is indicated following each aligning sequence. **B**: *cas-6* and *cg7229-5* share conserved elements that are unique (blue), repeat (red), or sub-repeat (gold) elements within the *cas-6* CSC (green underlined sequence indicates the mandatory element used to initiate the CSC database search). **C**: A *cg7229-5* CSC 12 species relaxed *EvoPrint*. Sequences that are present within *cas-6* CSBs are highlighted in the *cg7229-5* CSBs and color-coded to indicate their relative frequencies (see Fig. 4 for color code). **D**: *cg7229-5* CSC enhancer-reporter transgene expression analysis (*Gal4*-reporter mRNA in situ hybridization) reveals that, similar to the *cas-6* enhancer, the *cg7229-5* CSC functions as a late temporal window NB enhancer (embryo preparations as in Fig. 2A). Inset: Co-expression analysis reveals partial overlap between cells expressing *cas* mRNA (green) and those expressing the *cg7229-5* enhancer-reporter transgene mRNA (red; stage 11, dorsal whole-mount view).

**Fig. 4:**
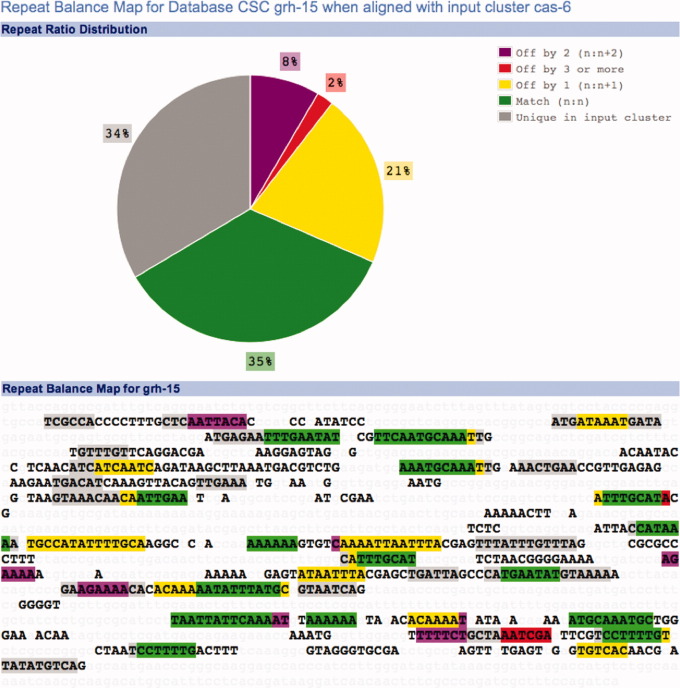
*cis*-Decoder analysis reveals that *cas-6* and *grh-15* CSCs share many sequence elements that are balanced in their copy number. Shown are a pie chart and a repeat balance map, both of which illustrate the relative copy number balance of shared elements between *cas-6* and *grh-15*. The repeat balance map of a relaxed *grh-15* CSC *EvoPrint* was highlighted to show comparative frequency of elements that are shared with the *cas-6* CSC. Green indicates balanced repeat element numbers between the two CSCs; yellow highlights repeats that are unbalanced by just one copy; purple, two copies; and red, three or more copies. Gray highlighted sequences are present just once in the *cas-6* CSBs. When uniquely shared sequences overlap repeat sequences, the repeat-ratio highlight color indicator is shown. When repeat elements overlap one another, the balance-ratio highlight of the longer repeat is shown, and when two repeats of equal size overlap, the more balanced repeat is highlighted.

After identifying RPS elements, the *cis*-Decoder algorithm searches the CSC database to discover CSCs containing these repeats. The search algorithm also allows for user supplied mandatory sequences, to identify enhancers that are regulated by sequence-specific DNA-binding factors or families of transcription factors. Once database CSCs are identified, the program carries out individual CSB alignments between the input CSC and the database CSCs (see below). Another set of algorithms then rates the individual database CSCs using the following similarity indices when compared to the input CSC: (1) A *repeat balance profile*, that assesses relative shared repeat copy numbers and weighs them according to the RPS length (shown as a pie chart and as a repeat balance map, which are accessible from the one-on-one alignment page; for examples see Figs. 3C, 4, 6B, 7A); (2) A *correlation coefficient*, which reflects the relative frequency of shared sequence elements between the input and database CSCs; (3) The *number of shared repeats* (full-length RPS elements and shorter elements contained within longer input repeats); (4) *Total number of shared elements* including RPS and uniquely shared sequences; (5) *Percent coverage* of aligning input sequences, which reflects the number of conserved bases in the database CSC that align with the input enhancer CSBs, normalized to the total number of conserved sequences in the database cluster; (6) The *number of user-specified required elements* present in the database CSC; (7) The *longest shared sequence* between the input and database CSCs (viewed at the *cis*-Decoder scorecard by placing the cursor on the sequence length number); and (8) The *total number of conserved bases* within the database CSC (see Table 1). To allow the user to focus attention on any one of the rating criteria, the CSCs can be sorted by any of the similarity indices in addition to sorting by CSC file name. Sorting by file name allows for the rapid identification of closely associated, neighboring CSCs that are structurally related to the input enhancer.

**TABLE 1:** *cas-6* CSC Database Search Results Showing Tested Clusters^a^

Cluster name	Correlation coefficient	Shared repeats	Total shared elements	Percent coverage	Required elements	Longest sequence	Conserved bases
cas-6	1.00	53	97	100	5	36	554
cg7229-5	0.63	20	44	56.88	4	11	320
vvl-14	0.56	26	65	58.63	3	11	498
nab-1	0.55	18	54	46.21	4	11	415
cg6559-28	0.52	29	58	54.38	3	11	521
cas-8	0.50	65	147	72.97	4	36	1,021
tkr-15	0.46	21	42	57.74	3	10	265
grh-15	0.44	41	96	64.98	5	9	554
vvl-43	0.42	42	93	60.03	3	11	633

a See Figures 2–7 for *in vivo cis*-regulatory activity.

### *cis*-Decoder Analysis of a *castor* Late NB Enhancer

To demonstrate the utility of *cis*-Decoder database search algorithms to identify tissue- and temporal-specific enhancers, we have used one of the late-temporal network NB enhancers (database CSC *cas-6*) that controls the embryonic expression of the gene encoding Cas, a zinc-finger transcription factor expressed during late embryonic CNS NB lineage development (Mellerick et al., 1992; Cui and Doe, 1992; Kambadur et al., 1998). Like endogenous *cas* mRNA expression, the *cas-6* enhancer activates reporter transgene expression in CNS NBs and ventral cord midline cells during embryonic stage 10 and in additional ventral cord and cephalic lobe NBs during stages 11–13 (Fig. 2A and Table 2; for *cas* mRNA and protein expression details see Mellerick et al., 1992; Kambadur et al., 1998). *EvoPrint* analysis reveals that the *cas-6* CSC is made up of 46 CSBs of 6 bp or more and contains 720 conserved base pairs in 1,613 bp of genomic sequence (Fig. 2B). Mutational analysis of the *cas-6* CSC via 5′ and 3′ deletions revealed that the entire cluster was required for full reporter activity (A. Kuzin, unpublished results). The *cas-6* CSC is located 392 bp 5&prime to the *cas* gene predicted transcriptional start site. As described above, one of the first steps in the *cis*-Decoder analysis is parsing CSBs from the input *EvoPrinted* enhancer in both forward and reverse directions, and then aligning the CSBs with one another (self-alignment) to discover RPS elements (Fig. 2C). More than 65% of the conserved bases in the *cas-6* CSBs were represented in RPS elements; an alignment revealed that these are either separate, adjacent, or overlapping each other (yellow-colored highlights in Fig. 2B). Core DNA-binding motifs for known transcription factors within CSBs are indicated in Figure 2B and C.

**TABLE 2:** Location, Structure, and Expression Dynamics of CSC Transgenes^a^

CSC name	Chromosome	CSC length (bp)	Conserved bases (bp)	Transgene Expression			Figures
				Embryo	Larva	Adult	
*cas-6*	3R	2,242	651	NBs^2^	None detected	None detected	2
*cg*7229-5	2R	849	367	NBs	None detected	None detected	3
*vvl-14*	3L	1,128	544	NBs	Not done	MB	5 and 9
*nab-1*	3L	1,012	369	NBs	Subset CL and VC NBs	MB	5 and 9
*cg6559-28*	3L	1,484	452	NBs	Many CL & VC NBs	MB, TmY	5 and 9
*cas-8*	3R	2,664	1331	NBs	Subset SOG neurons	None detected	5
*tkr-15*	2R	1,091	337	GMCs	Subset CL and VC NBs	None detected	5 and 9
grh-15	2R	1,376	621	NBs	Subset CL and VC NBs	None detected	5 and 9
*vvl-43*	3L	1,934	738	Ectoderm	Subset CL and VC neurons	SOG and optic lobe neurons	5 and 6
*sqz-11*	3R	1,082	427	NBs	Subset CL and VC NBs	None detected	5
*ct-14*	X	1,146	321	NBs	CL neurons and subset VC glia	None detected	5
*ct-3*	X	689	284	NBs	CL neurons and subset VC glia	None detected	5
*vvl-41*	3L	1,590	725	NBs	Subset CL and VC neurons	Subset of SOG neurons	5 and 7
*cg32264-76*	3L	833	263	None detected	Not done	Subset of CL neurons	S3

a Multiple, independent enhancer-reporter transgenes were tested for each CSC and all were integrated into the attP2 site on chromosome 3L at 68A4 via the Phi31 transgene integration method (Groth et al., 2004). NBs, neuroblasts; GMCs, ganglion mother cells; CL, cephalic lobes, VC, ventral cord; SOG, sub-esophageal ganglion; MB, mushroom body neurons; TmY, Trans-medullary Y neurons in optic lobe.

Prominent among the *cas-6* RPS elements are three 10mer repeat motifs [TTATGCAAAT], which contain a POU-homeodomain-octamer-binding site [ATGCAAAT] (Herr and Cleary, 1995). The highest copy number element [ATGCAAA], containing 7 of the 8 octamer motif sequences, was found 5 times (green underlined in Fig. 2C). It is considered a sub-repeat element, since there is only one instance of the heptamer in the CSBs that is independent of longer elements. Also present are multiple elements containing the core ATTA sequence for Antennapedia class homeodomain containing transcription factors (reviewed by Gehring et al., 1994). Also present in the RPS elements are two palindromic E-box sequences, CAATTG and CAGCTG (Murre et al., 1989), while three additional E-boxes are present in conserved non-repeated sequences. The *cas-6* enhancer CSBs also contains Hunchback and Cas core DNA-binding sequences (Fig. 2; Kambadur et al., 1998). Given that many of the *cas-6* RPS elements are novel sequences, they most likely contain additional binding sites for as yet uncharacterized transcription factors that modulate enhancer regulatory behavior.

### Searching for *cas-6* Related NB Enhancers

To identify database CSCs that share repeat and unique elements with the *cas-6* CSC, we initiated a search by first identifying CSCs that contained at least three copies of the ATGCAAA element. Although asking for a mandatory sequence is not required, the *cas-6* RPS table revealed that the highest copy number element, ATG CAAA, was present 7,208 times in the CSC database and 371 CSCs contained two or more of these elements. The *cis*-Decoder scorecard for this search revealed that the database contained 104 CSCs with 3 or more of this element (data not shown). Thus, we focused the search to this limited set of CSCs. Once these CSCs were identified, one-on-one alignments between the input and database CSBs were automatically performed to discover additional shared sequence elements. As expected, the highest scoring database CSC for most of the indices was *cas-6* itself (Table 1). Other high-scoring enhancers were considered as candidate late temporal network NB enhancers and were tested in enhancer-reporter transgenes (see below). For example, while *cg7229-5* scored highest for the correlation coefficient, other CSCs scored higher for each of the other metrics. Table 1 contains only a fraction of the database clusters in the actual readout (currently more than 100), since the database has been updated with additional CSCs after the initiation of the functional analysis of CSCs related to the *cas-6* enhancer.

Although the search required the hepamer sequence ATGCAAA to be present at least three times in the database CSC, most of the highest-scoring CSCs (both for correlation coefficients and shared RPS elements) contained at least three RPS elements with the full octamer motif [ATG CAAAT], including *cg7229-5, grh-15, vvl-41*, and *tkr-15* (Figs. 3B, 4B; data not shown). In addition, many of the CSCs that contained octamer motifs also shared, with *cas-6*, single or different combinations of bHLH E-box DNA-binding sites and repeated HOX-binding sites, including shared sequences flanking the core ATTA motif. An example of the one-on-one CSB alignment between *cas-6* and *cg7229-5* CSBs, discovered in this search, is shown in Figure 3A. Aligning *cas-6* CSB sequences are color-coded to represent *cas-6* RPS elements (red), truncated portions of the *cas-6* repeat sequences that we term sub-repeats (orange), and ≥6-bp sequences that are unique matches between *cas-6* and *cg7229-5* (blue). In many cases, different multi-copy repeats are nested within larger unique matches. For example, within the largest unique aligning sequence shown in Figure 3A, RPS elements corresponding to a HOX site overlap a POU-octamer site. We believe that this view of overlapping shared motifs represents a map of the substructure of an enhancer in terms of the transcription factor–binding sites that integrate multiple regulatory inputs.

*cis*-Decoder also generates lists sequence elements that are shared between the input and database CSC. For example, Figure 3B shows the complete output of repeat, sub-repeat, and unique matches between the *cas-6* and *cg7229-5* CSCs. Fifty-seven percent of the *cg7229-5* conserved sequences aligned with *cas-6* conserved sequences (Table 1 and Fig. 3C). In addition, *cis*-Decoder also identifies RPS elements within the input and database CSC that are not shared between the two CSCs, and these elements are also listed on the one-on-one alignment page (data not shown).

### Functionally Related NB Enhancers Share Balanced RPS Element Copy Numbers

The relative frequency of appearance of sequences in *cg7229-5* that correspond to *cas-6* RPS elements is shown by color-coded highlights (Fig. 3C). We term this comparison a “repeat balance map,” a visual representation that illustrates the relative frequency of appearance of each of the shared motifs in the comparison between the input and database enhancers. Forty-six percent of the aligning bases within the *cg7229-5* CSC are present in the same ratio in the *cas-6* CSC. The predominance of green and grey highlights indicates that many of the shared elements in the two enhancers are present at equal frequency. Another example of a CSC identified in this search that shares balanced RPS elements with the input *cas-6* is the *grh-15* CSC (Fig. 4; Table 1), also a temporal network NB enhancer (see below).

To test the in vivo *cis*-regulatory activity of CSCs, we selected CSCs that contained both repeat and unique sequence elements found in the *cas-6* enhancer. The CSCs were selected based on rating criteria described above, as shown in Table 1. Enhancer-reporter transgene transformants for the individual CSCs were generated using the targeted φC31 integration system to ensure that the regulatory behavior for each was assessed in the same genomic environment (see Experimental Procedures section and Supp. Fig. S4, which is available online). Although not an exact match, the expression pattern of the *cg7229-5* enhancer transgene shares many of the expression dynamics of the *cas-6* enhancer-transgene (Fig. 3D; Table 2). As with *cas-6*, onset of *cg7229-5* expression is in a subset of midline cells and a single lateral NB at stage 10, and expression in subsequent stages closely matches, but is not identical to, expression of the *cas-6* reporter. The insert shows that *cg7229-5* reporter GFP expression overlaps but is not identical to that of *cas-6* red fluorescent protein reporter.

Many of the tested CSCs (Table 2 and discussed below) yielded detectable CNS expression and function as late temporal network CNS neuroblast enhancers (Figs. 3, 5; Table 2; data not shown). Eleven were expressed in late temporal network ventral cord NBs and three were expressed in other CNS precursors or neurons (Figs. 3, 5). Comparing these expression patterns to the *cas-6* reporter expression (Fig. 2), it is apparent that each functions as a late temporal network enhancer. An indication of the specificity of the search for *cas-6*-like enhancers is that the search did not identify early temporal NB enhancers (Brody et al., 2008; Kuzin et al., 2009), nor did it identify broadly expressed NB enhancers such as that of *deadpan* (Emery and Bier, 1995).

**Fig. 5:**
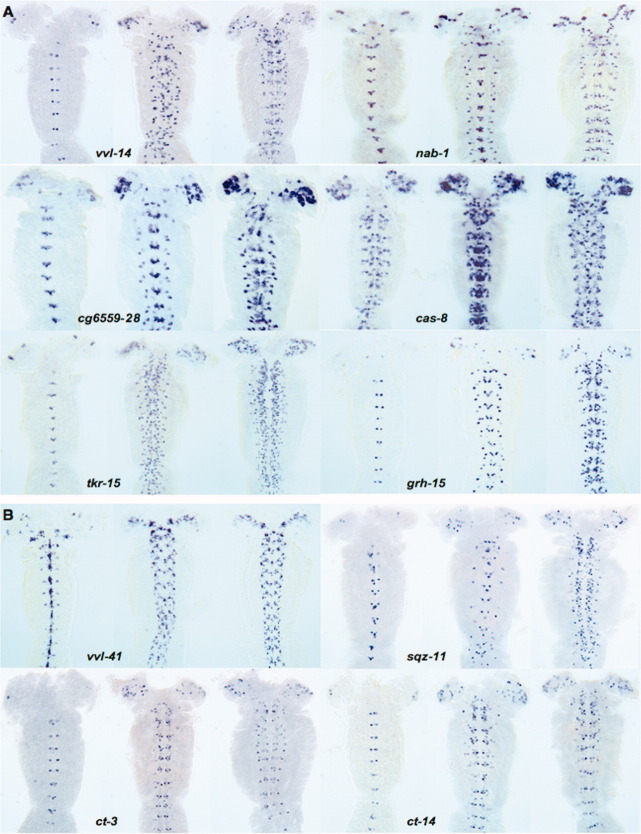
Identification of novel embryonic neural precursor cell enhancers based on their shared repeat sequences with other known neural enhancers. **A**: Like the *cg7229-5* enhancer (Fig. 3), additional database CSCs (Table 1) were identified that share balanced repeat sequences with the *cas-6* enhancer, and they also function as late NB sub-lineage enhancers. Many identified CSCs are adjacent to known NB expressed genes (*vvl, nab, cas, tkr*, and *grh*). **B**: Additional late sub-lineage neural precursor cell enhancers were also identified in *cis*-Decoder CSC database searches using CSBs from different NB enhancer CSCs as input (*vvl-41* and *sqz-11*, identified via the *cas-8* CSBs; *ct-3*, using the *pdm-2* gene NB enhancer CSBs; Berman et al., 2004); and *ct-14*, using the *cg6559-28* CSBs (Fig. 5A). Shown are dissected fillets of whole-mount-stained embryos, stages 10–12 (left to right, respectively, anterior up).

Although the *cas-6*-related enhancers are active in overlapping neural precursor cells, each has its own unique *cis*-regulatory identity. Each has a different pattern of expression in subsets of NBs, GMCs, and/or nascent neurons. For example, three identified enhancers (*nab-1, CG6559-28*, and *tkr-15*) exhibit early expression in a subset of ventral cord midline cells, while *sqz-11* and *vvl-41* (identified using *cas-8* as the input CSC) exhibit onset in a larger number of midline cells while other enhancers do not activate reporter expression in the midline precursor cells (Fig. 5). The *cas-8* CSC activated reporter expression in many more precursors at stage 11 than any of the other reporter constructs. *tkr-15* is expressed in many cells at stage 11. Since these cells are too small to be considered NBs, they are most likely GMCs or nascent neurons. Comparing different transgene reporter expression patterns in lateral ventral cord cells at stage 11 reveals that for certain CSCs, in particular *sqz-11, ct-3*, [identified using the *pdm-2* NB enhancer as input (see Fig. 5B)], fewer lateral cells express, or they exhibit uniquely different spatial expression patterns. This is also true for *ct-14* (identified using combined *cas-6* and *CG6559-28* as input) and *vvl-41* (identified *cas-8* as input). *cas-6* and *cas-8* enhancers both drive reporter expression in overlapping subsets of cells that represent sub-patterns of endogenous *cas* expression (Figs. 2, 5 and data not shown).

Our studies also revealed that there is no apparent consistency in the ordering, overlap, or orientation of shared elements between functionally related enhancers. For example, RPS elements shared between *cas-6, cg7229-5*, and *grh-15* appear in unique contexts within each enhancer (Supp. Fig. S1). This lack of consistency in positioning of shared elements has also been noted in early sub-lineage NB enhancers (Brody et al., 2008).

### Unbalanced RPS Elements Indicate Different Enhancer Regulatory Behaviors

**Fig. 6:**
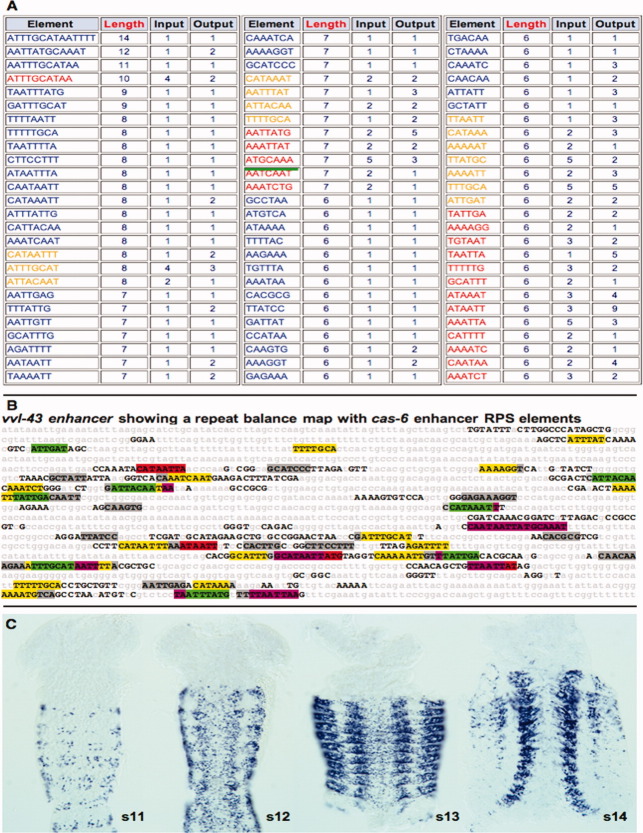
Enhancers that share unbalanced repeat elements between their CSCs carry out distinct regulatory functions. **A**: *cis*-Decoder alignments between the *cas-6* enhancer and *vvl-43* CSBs identified 93 different unique (blue), repeat (red), and shorter truncated-repeat (orange) sequence elements that were common to each CSC (green underline indicates the cas-6 repeat that was used to initiate the *cis*-Decoder CSC database search). **B**: The *vvl-43* CSC relaxed *EvoPrint* was highlighted to show repeat element frequencies relative to the *cas-6* enhancer (see color coding in Fig. 4). **C**: *vvl-43* CSC enhancer-reporter transgene expression analysis (*Gal4*-reporter mRNA in situ hybridization) reveals that, unlike the *cas-6* enhancer (Fig. 2A), *vvl-43* activates reporter expression in a subset of ectodermal cells during stage 11 and no reporter expression was detected in CNS NBs. Shown are filleted-flattened preparations of whole-mount-stained embryos, embryonic stages 11–14 (anterior up).

During the functional analysis of database CSCs that share RPS elements with *cas-6*, one of the CSCs, *vvl-43* (see Fig. 1B for *EvoPrint* profile), was found to share 92 RPS and unique sequence elements with *cas-6* (Fig. 6A). It did not, however, drive transgene reporter expression in NBs but activated expression instead in the embryonic ectoderm (Fig. 6C and Table 1). *cis*-Decoder analysis of the shared RPS elements revealed that the balance of PRS elements was markedly different between *cas-6* and *vvl-43* (Fig. 6B). Notable is the large number of conserved HOX motifs within *vvl-43* in comparison to *cas-6*. Expression of *vvl-43* in the embryonic ectoderm is segmental, and although temporally late, there is no embryonic CNS expression (Fig. 6C). Previous studies demonstrate that the *vvl*-encoded protein, a POU homeodomain factor, is expressed in the CNS and in the ectoderm of embryos, suggesting that *vvl-43* functions as an ectodermal enhancer for *vvl* expression (Anderson et al., 1995; also see figure 9A of Kambadur et al., 1998). The disparity of shared element frequencies between *cas-6* and *vvl-43* (Fig. 6B) is in marked contrast to the similarity of frequencies when comparing *cas-6* and *cg7229-5* (Fig. 3C). That lack of balance in shared element copy numbers between enhancers suggests that they may have different regulatory behaviors.

**Fig. 7:**
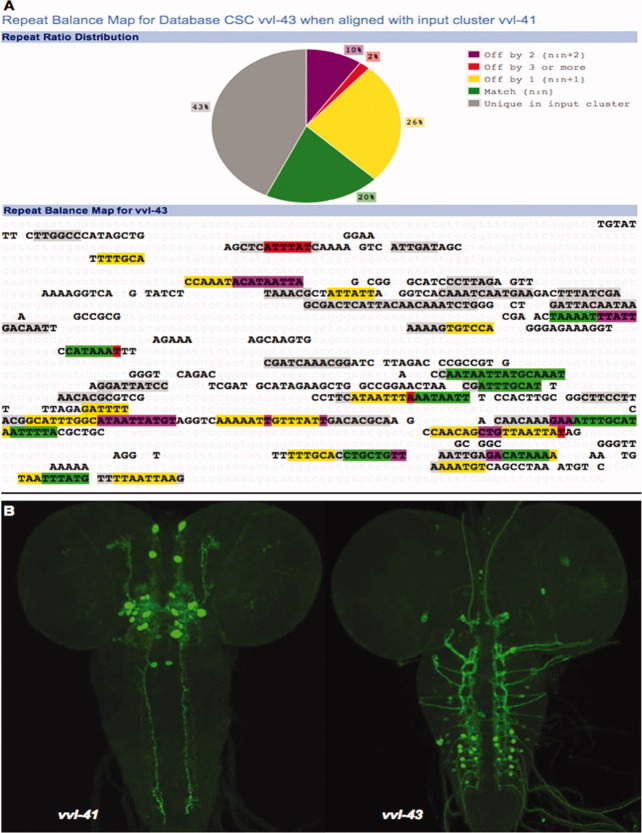
*vvl-41* and *vvl-43* enhancers exhibit an imbalance in copy number of their shared elements as evidenced by the low level of perfectly matched sequences. **A**: Shown are a pie chart and a *vvl-43* CSC repeat balance map that illustrate the relative copy number balance of shared elements between *vvl-41* and *vvl-43* (see Fig. 4 for ratio map color code). **B**: *vvl-41* and *vvl-43* CSCs function as larval neural enhancers that drive the expression of a membrane-bound GFP-CD8 reporter in different sets of CNS neurons. Shown are dissected cephalic lobes and ventral cords from wandering third-instar larva (dorsal views, anterior up).

Another example of how unbalanced RPS elements indicate functionally different enhancers can be seen in the comparative analysis of *vvl-41* with *vvl-43* CSCs (*EvoPrints* are shown in Fig. 1B). Like the previous comparisons to *cas-6*, the *vvl-41* and *vvl-43* CSCs share similar elements (Fig. 7); *vvl-41* shares 96 RPS and unique elements with *vvl-43* CSCs, and 68% of the *vvl-43* conserved sequences are covered by these shared elements (data not shown). Although these two CSCs have extensive overlap of shared elements, the repeat balance index and correlation coefficient reveal that their shared elements are not balanced in copy number (Fig. 7A and data not shown). Consistent with the imbalance in their shared elements, these enhancers displayed markedly different regulatory behaviors in the embryo (Figs. 5B, 6C). Nevertheless, these two enhancers drive reporter expression in different sets of larval neurons. Whereas most of the cells expressing the *vvl-41* reporter transgene are sub-esophageal ganglion interneurons, *vvl-43* enhancer drives reporter expression in a subset of ventral cord motor neurons (Fig. 7B). Thus the presence of identical elements in different clusters does not necessarily lead to similar regulatory behaviors, and comparing shared element copy-numbers has a better predictive value for determining enhancer behavior.

### *cis*-Decoder Searches Identify Novel Sequence Elements Present in Other Families of Functionally Related Enhancers

**Fig. 8:**
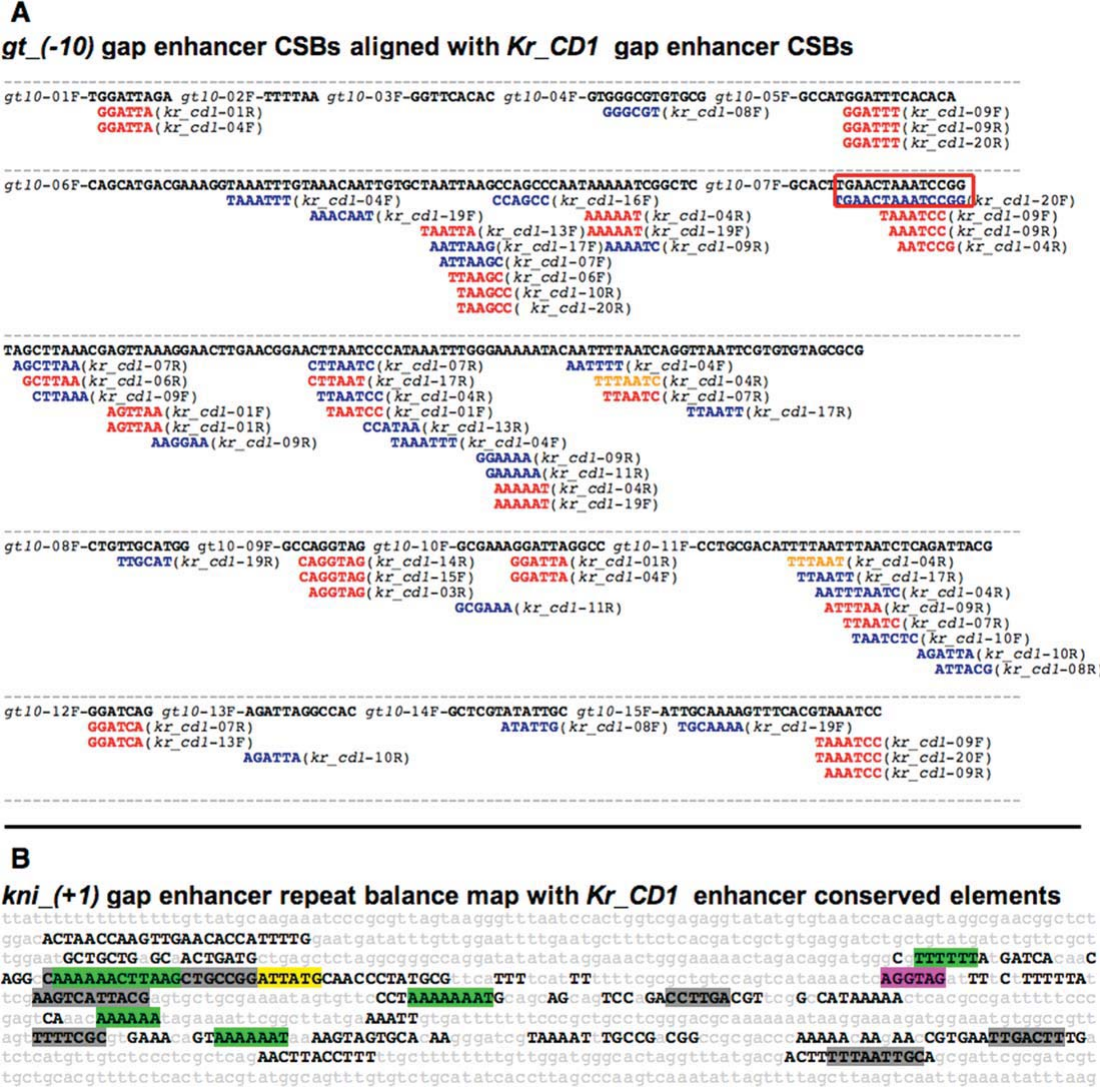
*cis*-Decoder CSC database searches identify shared conserved sequence elements among cellular blastoderm gap enhancers. **A**: CSB alignments between the *Krüppel* and *giant* gap enhancers (*Kr_CD1*, Hoch et al., 1992; *gt_10*, Schroeder et al., 2004) identify 42 distinct conserved sequence elements of 6 bp or greater that represent 55.62% of the conserved bases within the *gt_10* enhancer. The red-colored boxed 14-bp sequence corresponds to the characterized overlapping *Knirps* and *Bicoid* transcription factor bindings sites that are required for the wild-type *Kr_CD1* enhancer regulatory behavior (Hoch et al., 1992). **B**: The *knirps* gap enhancer CSC (*kni_(+1*); Schroeder et al., 2004) relaxed *EvoPrint* was highlighted to show shared RPS and unique element frequencies present in the *Kr_CD1* gap enhancer (see color coding in Fig. 4 for RPS balance index).

To further test the ability of *cis*-Decoder database searches to identify different families of functionally related enhancers and to compare our search protocols to other enhancer search algorithms, we initiated database searches with different well-characterized enhancer types. Using the *Krüppel* gap enhancer *Kr_CD1* (Hoch et al., 1990), we identified the *giant gt_(−10*) enhancer (Schroeder et al., 2004) (Fig. 8A). Besides sharing HOX sites with different flanking bases (Fig. 8A), the two enhancer CSCs also share a 14-bp sequence, TGAACTAAATCCGG (see boxed sequence in Fig. 8A). Remarkably, this 14-bp element within the *Krüppel* enhancer was identified as a site of competitive binding by the activator Bicoid and the repressor Knirps transcription factors (Hoch et al., 1992). The conservation of interlocking or overlapping docking sites for Bicoid and Knirps within both of these gap enhancers supports the contention that large CSBs (containing 7 to 10 bp or more) most likely function as the point of integration of multiple transcription factors in the regulation of enhancer behavior.

Our search using the *Kr_CD1* also identified the *kni_(+1*) intronic gap enhancer (Schroeder et al., 2004). Shared sequence motifs between *Kr_CD1* and *kni_(+1*) include multiple polyA/polyT motifs, presumably targets of Hunchback, that are found in even balance (five copies) between the two enhancers (Fig. 8B). Other shared sequences include several HOX-binding sequence elements.

Previous work has shown that many segmentation genes utilize multiple enhancers that regulate gene expression in nearly identical patterns (reviewed by Hobert, 2010). These enhancer pairs have been termed (1) primary enhancers, found closely associated with the transcriptional start site, and (2) “shadow” enhancers, found at a distance from the structural gene. Starting with the primary *vnd* ventral neuroectoderm enhancer CSC (Hong et al., 2008), a *cis*-Decoder search identified its shadow enhancer based on the balanced copy number appearance of its RPS elements and uniquely shared sequences (Supp. Fig. S2; and data not shown). In addition to other shared elements, both of these enhancers contain 2 copies of the CACATGA bHLH motif, which matches the optimal DNA-binding site for the transcriptional regulator Twist (Ozdemir et al., 2011).

We next tested the *cis*-Decoder search algorithms to see if it would be possible to detect enhancers regulated by Notch signaling (Nellesen et al., 1999). Previously identified Notch-targeted enhancers include those associated with the *E(spl*) complex genes. Multiple alternative binding sites within these enhancers have been identified for Suppressor of Hairless [Su(H)], the transcription factor utilized by the Notch pathway (Bailey and Posakony, 1995; Castro et al., 2005). We initiated a *cis*-Decoder search with one of the CSCs (*Espl-1*) to discover other similarly structured CSCs, using as required sequences a single Su(H)-binding site (TGGGAA) and a single bHLH-binding site (CAGCTG). This search resulted in 101 database hits, including CSCs from known Su(H) targets *m2, m6*, and *m*γ (Castro et al., 2005) as well as putative enhancers for the neural determinants *Dichaete, deadpan, nervy, tailless, castor, Fps85D, Notum*, and *extra macrochaetae* (data not shown). In addition, searching with the Notch-targeted *deadpan* NB enhancer (San-Juán and Baonza, 2011; *cis*-Decoder CSC *dpn-3*), that contains two alternative Su(H)-binding sites (GTGAGAA; Bailey and Posakony, 1995; Lecourtois and Schweisguth, 1995; Nellesen et al., 1999), we identified other putative Notch pathway targeted enhancers: *CG7229-5, cas-8*, a *HLHm*β-associate CSC (HLHmbeta-2), and the *m4* PNS enhancer (Nellesen et al., 1999). Thus, *cis*-Decoder searches can identify functionally related enhancers that regulate gene expression during different phases of development and in different tissues.

### Many Enhancers Regulate Gene Expression During Multiple Phases of Development

**Fig. 9:**
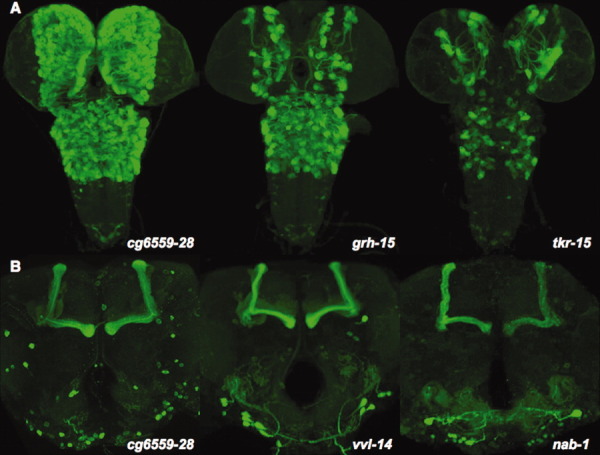
Many NB enhancers that regulate gene expression during embryonic CNS development also activate gene expression during adult development and in the adult nervous system. **A**: During third-instar larval development, the *cg6559-28, grh-15*, and *tkr-15* CSC enhancer-*Gal4* driver transgenes activate membrane-bound GFP-CD8 tagged transgene expression in sub-regions of the cephalic lobes and in thoracic ventral cord neural precursor cells. Shown are dorsal views of dissected CNS preparations from wandering third-instar larva (anterior up). **B**: In the adult brain, the *cg6559-28, vvl-14*, and *nab-1* enhancers drive GFP-CD8 reporter expression in neurons whose cell bodies reside in the mushroom body calyxes and in different regions of the sub-esophageal ganglion. Shown are confocal, optical sections of GFP immunostained adult brains (frontal views) at the level of the mushroom bodies and the sub-esophageal ganglion.

Each of the embryonic NB enhancers identified above were also tested for regulatory activity during later stages of development, and many were observed to activate transgene reporter expression in the third instar larva and/or adult CNS. Three of the tested enhancer transgene reporters, *cg6559-28, grh-15*, and *tkr-15* exhibited expression in a similar pattern within brain neural precursor cells, thoracic neuromeres and posterior neural precursors of the thirdinstar larva CNS, while the *cas-6* and *cas-8* enhancers were not active in larvae (Fig. 9A; Table 2; and data not shown). The *ct-3* and *ct-14* CSCs drove expression in small subsets of neurons in the sub-esophageal ganglion and in the ventral cord abdominal neuromeres (data not shown). Additionally, *nab-1* expression was similar to that of the *dnab*^*e310*^ enhancer-trap expression in third-instar larvae CNS (Clements et al., 2003; data not shown). In the adult, many of the enhancers were expressed in a subset of central brain neurons, and in the optic lobe. Specifically, *cg6559-28, vvl-14*, and *nab-1* reporters were expressed in the mushroom body (Fig. 9B). While *cas-6* was not expressed in the adult brain, *cas-8* reporter expression was detected in the ellipsoid body in a pattern similar to *cas* adult expression (Hitier et al., 2001; data not shown). In addition to analyzing the 14 CSCs listed in Table 2, we also examined the embryonic and adult reporter expression of another 60 CSCs, chosen by a variety of criteria. Many of these activate transgene reporter expression in both the embryonic and adult CNS (data not shown). Given the fact that CSC sub-regions of these multiuse enhancers have not been tested for reporter activity, we cannot rule out the possibility that different regions within the cluster have autonomous functions and represent discrete enhancers. However, our functional analysis of the *nerfin-1* NB enhancer and the *cas-6* enhancer CSCs has revealed that full enhancer function requires the complete cluster (Kuzin et al., 2009 and unpublished experiments). The *EvoPrinter* algorithm provides a methodology for testing for the close apposition of independent enhancers (Kuzin et al., 2009).

### Dissecting P-Element Enhancer-Trap Line Expression Patterns

Previous *cis*-regulatory analysis of genomic regions flanking enhancer-trap insertion sites has revealed the basis of enhancer-trap expression in terms of flanking endogenous enhancers regulating P-element reporter transgenes (O'Kane and Gehring, 1987). P-element Gal-4 enhancer-trap lines have been used extensively to drive transgene expression during development (reviewed by Hummel and Klämbt, 2008). Although this approach has been of great utility, many of the Gal4 driver lines are of limited use due to their broad expression patterns, which is most likely due to multiple tissue/temporal specific enhancers regulating Gal4 transgene expression. In addition to the discovery of new enhancers, *cis*-Decoder tools can be used to identify and differentiate between enhancers that flank the insertion site of P-element enhancer-trap constructs. For example, the enhancer-trap Gal4 line c492a activates UAS-transgene expression in a subset of neurons within the adult mushroom body, in the antenna lobe, and in a subset of neurons in the central brain (Armstrong and Kaiser, 1997; Flytrap web site: http://www.fly-trap.org/). The c492a P-element insertion site was localized to the 4th intron of an uncharacterized gene, *cg32264* (see Supp. Fig. S3A). *cis*-Decoder analysis of CSCs in the vicinity of the insertion site revealed a candidate CSC, *cg32264-76*, with sequence properties suggestive of a neural enhancer. RPS analysis revealed that *cg32264-76* contains two extended octamer motifs [TTATGCAAAT] (Supp. Fig. S3B). The *cg32264-76* reporter expression pattern corresponds to a subset of neurons marked by the c492a enhancer-trap GAL4 reporter. The adult expression pattern included a large neuron that extends dendrites into the optic lobe (Supp. Fig. S3C and Table 2). This suggests that the combined *EvoPrinter* and *cis*-Decoder analysis will help in the identification of specific enhancers to further refine transgene expression.

### Criteria for Identifying and Evaluating Related CSCs

Although each of the *cis*-Decoder scorecard indices provides useful information in judging the relationship of the input enhancer to database CSCs, we have found the repeat balance index and the correlation coefficient (see criteria 1 and 2 above) are more accurate indices when searching for functionally related enhancers, since they take into account not only the number of shared elements but also the RPS copy number balance between the input enhancer and database CSC. The percent alignment coverage is likewise an important indicator of the relationship between the input and database CSCs. Thus, sorting the scorecard by the repeat balance index or by the correlation coefficient increases the likelihood that functionally related enhancers rank at the top of the list. For example, all of the late temporal NB enhancers identified in this study had repeat balance index scores of greater than 1.0, correlation coefficient rankings of above 0.4, and percent coverage of ≥40%.

To estimate the number of false-positive predictions and functionally related enhancers that were missed in *cis*-Decoder searches, we used the *cas-6* as the input enhancer (for search conditions see above). The search returned 111 database hits, of which 27 that shared many repeat elements with *cas-6* were tested for enhancer activity in flies. Of these, 12 proved to be late temporal network enhancers, with each being expressed in a different subset of midline, brain, and/or ventral cord neuroblasts. Eleven were expressed exclusively either in adult brain, larval precursors, or in embryonic neurons, and four were considered negative, since their reporter expression was undetectable or found in other tissues other than the nervous system. As for enhancers that were missed in the search, we have identified late temporal network enhancers that do not contain three or more complete or partial octamer sequences, or do not score highly using *cas-6* as input. The low-scoring enhancers included *sqz-11* and *vvl-41*, which were discovered using *cas-8* as the input CSC (mentioned above). Likewise, *ct-3* and *ct-14* did not contain three octamer sequences, and they also proved to be late temporal network NB enhancers. Finally, we have identified five other late temporal network enhancers that do not contain octamer motifs but do contain other repeated elements found in late temporal network enhancers (data not shown). It is clear from these results that a search for enhancers using a mandatory sequence, such as the octamer motif, is insufficient to detect the full genomic repertoire of late temporal network enhancers. To identify as many functionally related enhancers as possible, multiple database searches using different search criteria, are recommended. Our current understanding of the role of octamer motifs in conferring temporal gene expression is incomplete, in that we are unable to fully distinguish between embryonic late temporal network enhancers, and octamer-site rich larval or adult brain enhancers. Nevertheless, the fact that only four of the 27 clusters tested were not expressed in the CNS, speaks to the efficacy of *cis*-Decoder search algorithms in detecting neural enhancers.

Ideally, it would be useful to make direct comparisons of the *cis*-Decoder algorithm with other web-based tools for discovery and analysis of *cis*-regulatory elements. However, not all search programs use evolutionary comparisons, and those that do use different levels of evolutionary divergence to identify conserved sequences in enhancers. The comparative analysis of enhancer discovery programs nevertheless points to factors present in various computational formats that appear to be important for successful *cis*-regulatory element prediction (discussed in Su et al., 2010). These include sequence conservation between related species, motif clustering, and availability of prior information on the presence of known transcription factor–binding sites. In this context, combined use of *cis*-Decoder methodology with Chip-Seq data, that shows occupancy of *cis*-regulatory modules by specific transcription factors (Zinzen et al., 1999; Wilczyński and Furlong, 2010), will improve identification of functional motifs within enhancers that are bound by specific transcription factors, and resolves additional functionally important flanking sequences. The libraries of repeat and uniquely shared sequences generated by *cis*-Decoder are useful for sub-structural analysis of enhancers; for example, discovery of the unique element shared by *Krüppel* and *giant* gap enhancers demonstrates the ability of *cis*-Decoder to reveal combinatorial interactions by analysis of blocks of conserved sequences. Other aspects of *cis*-regulatory biology will also be relevant; for example, the configuration of the chromatin as detected by DNase1 hypersensitivity indicates accessibility of enhancer sequences to transcriptional regulators (reviewed in Suganuma and Workman, 2011). The knowledge of chromatin state is invaluable for prediction of enhancer activity, and information concerning specific CSCs can be accessed via the UCSC browser.

Efficacy of *cis*-Decoder in predicting enhancers can be compared to a study that used known *cis*-regulatory modules to develop a training set of computationally predicted transcription factor–binding sites to predict genomic *cis*-regulatory modules (Rouault et al., 2010). That study predicted neural expression of the same *cg7229* enhancer that was identified using *cis*-Decoder (Fig. 3). Likewise an algorithm known as Ahab, which uses transcription-factor-binding-site information for known regulators of cellular blastoderm enhancers, successfully predicted the *gt_(−10*) and *kni(+1*) gap enhancers (Schroeder et al., 2004) that also scored highly in our search using the *Kr_CD1* gap enhancer as the input CSC (Fig. 8). It is important to point out that *cis*-Decoder search protocols make direct use of CSC information for enhancer prediction, while other resources, such as Genome Surveyor (Kazemian et al., 2011), use site conservation as a criterion, but do not provide information to infer enhancer boundaries. Given that multiple enhancer prediction programs that employ different search criteria are available, it would be advisable to employ several discovery programs (summarized by Su et al., 2010) before settling on a final list of candidate genomic regions for analysis in enhancer-reporter transgenic studies.

## CONCLUSIONS

We have generated a *Drosophila* genome-wide database of evolutionarily conserved DNA sequences that allows for discovery of functionally related enhancers. A *cis*-Decoder search identifies database CSCs that share balanced conserved sequence elements with an input enhancer. No prior information about the functional significance of DNA sequences within enhancers is required to identify other related enhancers. The database provides an inventory of conserved repeat sequences within CSCs and enables comparison between input and database CSCs by various metrics that allow the user to judge CSC similarity. Starting with a temporally restricted NB enhancer, we have shown that *cis*-Decoder can successfully identify other similarly regulating enhancers, and we also demonstrate how other functionally distinct enhancer families can be identified.

Our comparative analysis of enhancers described in this report and an additional 60 enhancers, have yielded the following observations considering enhancer structure and behavior: (1) Functionally related enhancers can be identified based on their balanced copy numbers of shared conserved repeat elements. (2) Enhancers that have extensive shared conserved sequence elements (often >60%), but do not have balanced shared repeat copy numbers, may display significantly different regulatory behaviors. (3) Shared repeat and unique elements between functionally related enhancers are not found in any fixed order or orientation. (4) Similarly regulating families of enhancers need not share specific sets of conserved sequence elements, since different enhancers can accomplish the same regulatory behavior with different but overlapping sets of conserved elements. (5) Enhancers that share conserved repeat elements and perform related *cis*-regulatory functions also contain unique sets of repeat elements that are only partially shared with other related enhancers.

Our observations have revealed that *Drosophila* CNS developmental enhancers are highly complex, based on their conserved sequence composition, and many have proven to be multifunctional. The observed complexity of enhancers, specifically with regard to multi-copy repeat motifs, also suggests that enhancer function is realized through a complex process involving combinatorial interactions among many factors and cannot be easily explained by single activator/repressor transcription factor switches. In addition, the fact that functionally diverse enhancers can display such extensive overlap in their conserved sequences underscores the combinatorial complexity of *cis*-regulation (also see Southall and Brand, 2009). Because of the lack of fixed order and orientation of shared elements between related enhancers, only the alignment flexibility of the *cis*-Decoder CSB aligner can rapidly detect the extent and makeup of shared conserved sequences between different enhancers. Until now, enhancer boundaries have, for the most part, been resolved by reporter transgene deletion analysis. The addition of evolutionary clustering of conserved sequences to this identification process will aid in enhancer identification and allow for an assessment of their structure and spatial constraints. *cis*-Decoder algorithms also allow one to generate libraries of conserved sequence elements that are shared among enhancers; this dataset will be useful for understanding the combinatorial complexity of tissue-specific gene regulation.

## EXPERIMENTAL PROCEDURES

### Conserved Sequence Cluster *EvoPrints*

*EvoPrint* conditions for identifying the database CSCs are described in the Results and Discussion section. For the CSCs used as examples in the text and figures, relaxed *EvoPrints* were prepared using *D. melanogaster* DNA as the reference sequence and 11 orthologous DNA sequences from the *D. simulins, D. sechellia, D. yakuba, D. erecta, D. ananassae, D. pseudoobscura, D. persimilis, D. willistoni, D. virilis, D. mojavensis*, and *D. grimshawi* species.

### Enhancer Search Protocol

*cis*-Decoder (http://cisdecoder.ninds.nih.gov/public.do) programs consist of an integrated set of search and alignment algorithms that help discover conserved sequence elements that are shared between similarly regulated enhancers. The following is a description of the sequential steps and accompanying algorithms used by *cis*-Decoder protocol to identify repeat and palindrome elements within the input cluster and to scan a genomic CSC database for other CSCs that contain the conserved sequence elements present in the input CSC.

The *Drosophila* CSC database is based on relaxed *EvoPrints* (Yavatkar et al., 2008; http://evoprinter.ninds. nih.gov/) of >90% of the eukaryotic genome. Clusters were identified and named using *EvoPrint* cutter, an Image J macro written by Wayne Rasband of NIMH. CSBs were extracted in forward and reverse directions, a database repository was populated with CSC details including a list of CSBs, and records of repeat and palindromic elements and their number within each cluster as well as across all the clusters in the database.

Upon input of a user-provided CSC, *cis*-Decoder extracts and annotates the CSBs in both forward and reverse directions and discovers repeat and palindromic sequences within the input CSC. The system also invokes a database search to find accruals for each repeat that is found in database repository along with a display of the number of database clusters containing more than one copy of each repeat. The user sets search constraints to limit search to specific list of repeat motifs as well as sequence type (non-coding, coding, or 3′ UTR).

*cis*-Decoder then searches the database repository for clusters that conform to the search constraints. An alignment between the input and database cluster is generated that shows alignments to input enhancer multi-copy repeat motifs along with input enhancer repeats that were excluded from the database search, unique alignments, and database cluster repeat sequences found as a subset of the unique alignments.

Various alignment scores are computed to rank the database CSCs; scores include the RPS Balance Index (a measure of the relative balance of the perfectly matched shared RPS elements to the shared RPS elements that are not matched in frequency between input and database CSC), the Pearson Correlation Coefficient (a measure of the strength of a linear relationship between the relative occurrence of repeat and unique matches in the input vs. the database cluster), Number of Shared Repeats, Total Shared Elements (including repeat, palindromic, and uniquely shared elements), Percent Coverage (the percent of conserved bases in the database CSC aligning to RPS and unique matches in the input enhancer CSBs), Number of Required Repeats (set as a search criterion), Longest Shared Sequence, and the number of Conserved Bases in the database CSC. *cis*-Decoder then generates lists of shared sequences of input and database CSCs, and generates a “repeat balance map” visual representation of the relative frequency of appearance of the RPSs in the input versus database CSC (see Fig. 4 legend for details; when repeats overlap, the balance ratio designation of the longer repeat is indicated and when two repeats of equal size overlap, the more balanced ratio is indicated). In addition to the repeat balance map, a representation of the balance of shared elements within the database CSC in comparison to the input is shown in the form of a pie chart.

### Enhancer-Reporter Transgene Construction

Fragments for cloning were generated by standard PCR protocols. Primer sequences are provided in Table S1. PCR-amplified genomic fragments were cloned into Invitrogen pCRII-TOPO vector, sequenced for verification of the insert, and recloned into a site-specific integration vector, Bullfinch (Fig. S3), consisting of a modified pCa4B vector (Markstein et al., 2008) with an inserted polylinker site, minimum Heat shock protein 70 promoter (from the pRed H-Stinger vector; Barolo et al., 2004), Gal4 ORF (from *S. cerevisiae*), and SV40 3'UTR (from the pRed H-Stinger vector; Barolo et al., 2004). Bullfinch Vector map is found in Supp. Fig. S4. Details of the cloning steps and vector sequence are available upon request.

### *Drosophila* Stocks and P-Element Transformations

Third chromosome site-specific P-element integration transformants were generated in the y, w; y+[attp2] strain as previously described (Groth et al., 2004: Markstein et al., 2008) using our Gal4 site-specific vector (see above). Embryo Gal4 mRNA in situ hybridizations were performed on multiple independent transformant lines for each construct to assure reproducible expression.

### Embryo Transgene Reporter Expression Localization

Embryo collection and fixation were performed according to the procedures described by Patel (1994). For in situ hybridization, riboprobes were prepared from a PCR-amplified Gal4 ORF within the Gal4 Bullfinch vector. Roche (Indianapolis, IN) DIG RNA Labeling Mix protocol was used, and staining was visualized using anti-FITC Fab fragments coupled to alkaline phosphatase. Transgene-reporter expression for each of the enhancers was examined in at least two independent transgene-reporter transformant lines. *cas* mRNA transcripts were detected by in situ hybridization using a *cas* ORF digoxigenin probe generated from a PCR amplified genomic fragment. More detailed protocols for embryo processing and in situ hybridization are available upon request. After whole-mount in situ hybridization, embryos were filleted, viewed in 70% glycerol with 30% phosphate-buffered saline (PBS), and photographed using a Nikon (Melville, NY) microscope equipped with Nomarski (DIC) optics. Embryo developmental stages were determined by morphological criteria (Campos-Ortega and Hartenstein, 1985).

### Immunohistochemistry and Confocal Imaging of Larval and Adult Brains

GAL4 expression in the larval brain and CNS of wandering larvae was analyzed using mCD8::GFP (Lee and Luo, 1999) as reporter. Brain dissection, immunohistochemistry, and confocal imaging [using a Zeiss LSM710 and Plan-Apochromat objective 10× (n.a, = 0.45)] were performed as described previously (Lee and Luo 1999). For immunohistochemistry, rabbit anti-GFP (1:1,500, Invitrogen, San Diego, CA) and Alexa 488 goat anti-rabbit IgG (1:1,000, Invitrogen) were used to enhance the GFP signal. Serial optical sections (1,024×1,024 pixel resolution) were taken at 2-μm intervals along the dorso-ventral axis. The confocal image stacks were analyzed using ImageJ software (NIH, Bethesda, MD).

For each genotype, at least three adult flies of mixed genders were collected 3 to 5 days after eclosion and used for immunohistochemistry and imaging. Brain dissection, immunohistochemistry, and confocal imaging [using a Zeiss LSM510 META and plan Neofluar objective 40× (n.a, = 1.3)] were performed as described previously (Gao et al., 2008). For immunohistochemistry, rabbit anti-GFP (1:300, Torrey Pines Biolabs, East Orange, NJ) and Alexa 488 goat anti-rabbit IgG (1:250, Molecular Probes, Eugene, OR) were used to enhance the GFP signal. Serial optical sections (512×512 pixel resolution) were taken at 1-μm intervals along the rostro-caudal axis. The confocal image stacks were analyzed using Imaris (Bitplane, Zurich, Switzerland) software.

## Supplementary material

Supporting Information Figure 1. RPS elements that are shared among functionally related enhancers are not found in any common order or orientation within the different enhancers. Shown are relaxed D. melanogaster genomic DNA EvoPrints (12 drosophilids) of the functionally related cas-6, cg7229-5 and grh-15 late temporal NB enhancers. Highlighted with different colors are 8 RPS elements of 6 bp or greater in length that are present in each of the three enhancers. Note, many of the RPS elements overlap other elements but in different and arrangements within the enhancer CSBs.

Supporting Information Figure 2. Primary and shadow enhancer CSBs share common balanced sequence elements. Shown are relaxed D. melanogaster genomic DNA EvoPrints (12 drosophilids) of functionally related enhancers that regulate vnd gene expression in overlapping expression patterns within the embryonic neurogenic ectoderm (described by Hong et al., 2008; boxed sequences indicated enhancer-reported tested DNA). Highlighted CSB sequences represent shared ≥6-bp unique or repeat sequence elements between the primary and shadow enhancer CSCs. Gray highlighted sequences are present once in the vnd primary enhancer CSC (note; when uniquely shared elements overlap repeat sequences, the repeat-ratio highlight color is shown). Green indicates balanced repeat numbers between the two CSCs; and yellow highlights repeats that differ in frequency by just one copy. When repeat elements overlap, the balance-ratio highlight of the longer repeat is shown; when two repeats of equal size overlap, the more balanced repeat highlight is shown.

Supporting Information Figure 3. cis-Decoder analysis of sequences flanking P{Gal4} insertions identifies a flanking enhancer that regulates the P-element transgene. A: A linear cartoon of the CG32264 predicted gene locus on chromosome 3L. Dark-blue boxes indicate ORF containing exons, the P{Gal4}c492a (Armstrong and Kaiser, 1997) integration site is indicated as a red-colored triangle, and the position of the cg32264-76 CSC is highlighted as a light-blue box. B: A relaxed EvoPrint of the cg32264-76 CSC genomic region (boxed sequence indicates the enhancer-reported tested DNA). CSB sequences that span repeat elements are highlighted in light purple color. One-on-one CSB alignments of the cg32264-76 CSC, utilizing cis-Decoder, identified 14 distinct repeat elements that are ≥6 bp. A total of 31 repeat elements cover 57.46% of the conserved sequences. Highlighted with different colored bases are underlined sequences that correspond to the core DNA-binding sites of different transcription factors (homeodomain, ATTA-red; POU domain, ATGCAAAT-green; bHLH, CANNTG-brown: Hunchback/Castor, TTTTT/AT-blue; and PBX sites, TGAT-teal). C: In the adult brain, the cg32264-76 CSC enhancer-Gal4 driver transgene activates GFP-CD8 tagged transgene expression the central brain, sub-esophageal ganglion, and in optic lobe neurons. Shown is a confocal, optical section of an adult brain (frontal view).

Supporting Information Figure 4. Bullfinch, a site-specific integration vector, consists of a modified pCa4B vector (Markstein et al., 2008) with an inserted polylinker site (A. Kuzin), minimum Heat shock protein 70 promoter (from the pRed H-Stinger vector; Barolo et al., 2004), Gal4 ORF (from S. cerevisiae), and SV40 3′UTR (from the pRed H-Stinger vector; Barolo et al., 2004).
