# Supplementary figures and images for: Use of a Drosophila Genome-Wide Conserved Sequence Database to Identify Functionally Related cis-Regulatory Enhancers
Source: Dev Dyn. 2011 Aug 30;241(1):169–89. doi: 10.1002/dvdy.22728 (PMC3243966; doi:10.1002/dvdy.22728)

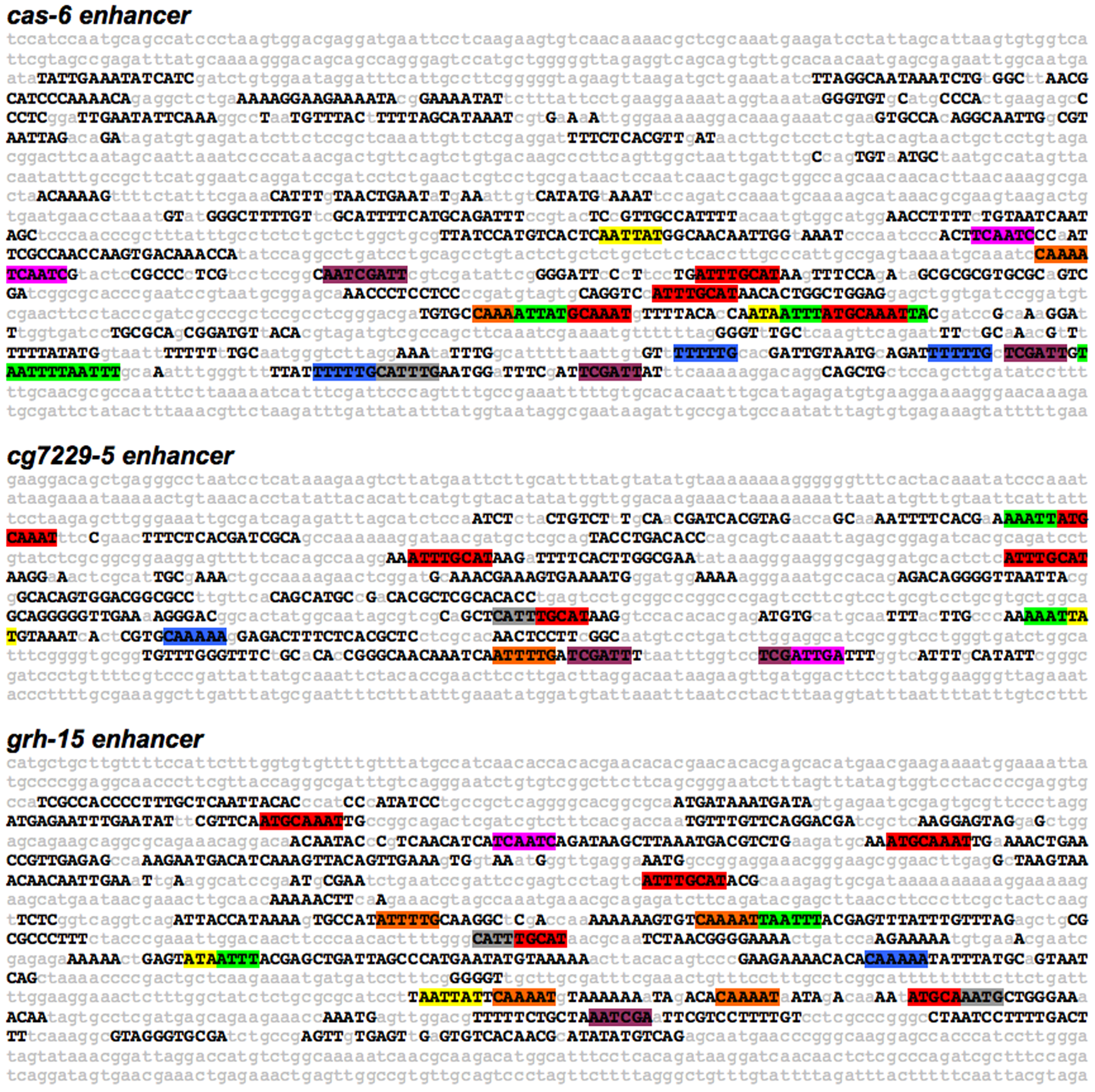

Supplement: Supplementary file 1 [file dvdy0241-0169-SD1.tif]

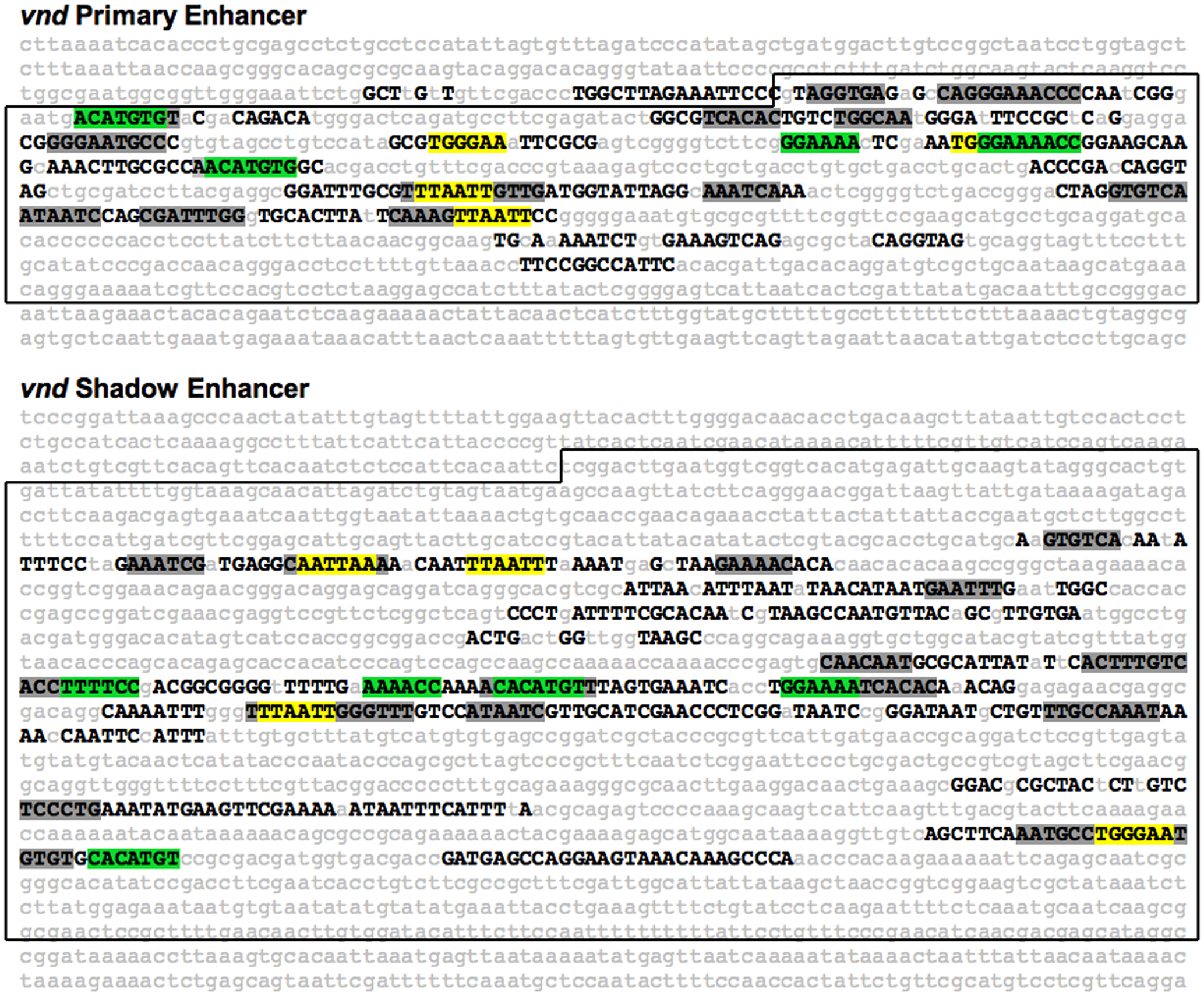

Supplement: Supplementary file 2 [file dvdy0241-0169-SD2.tif]

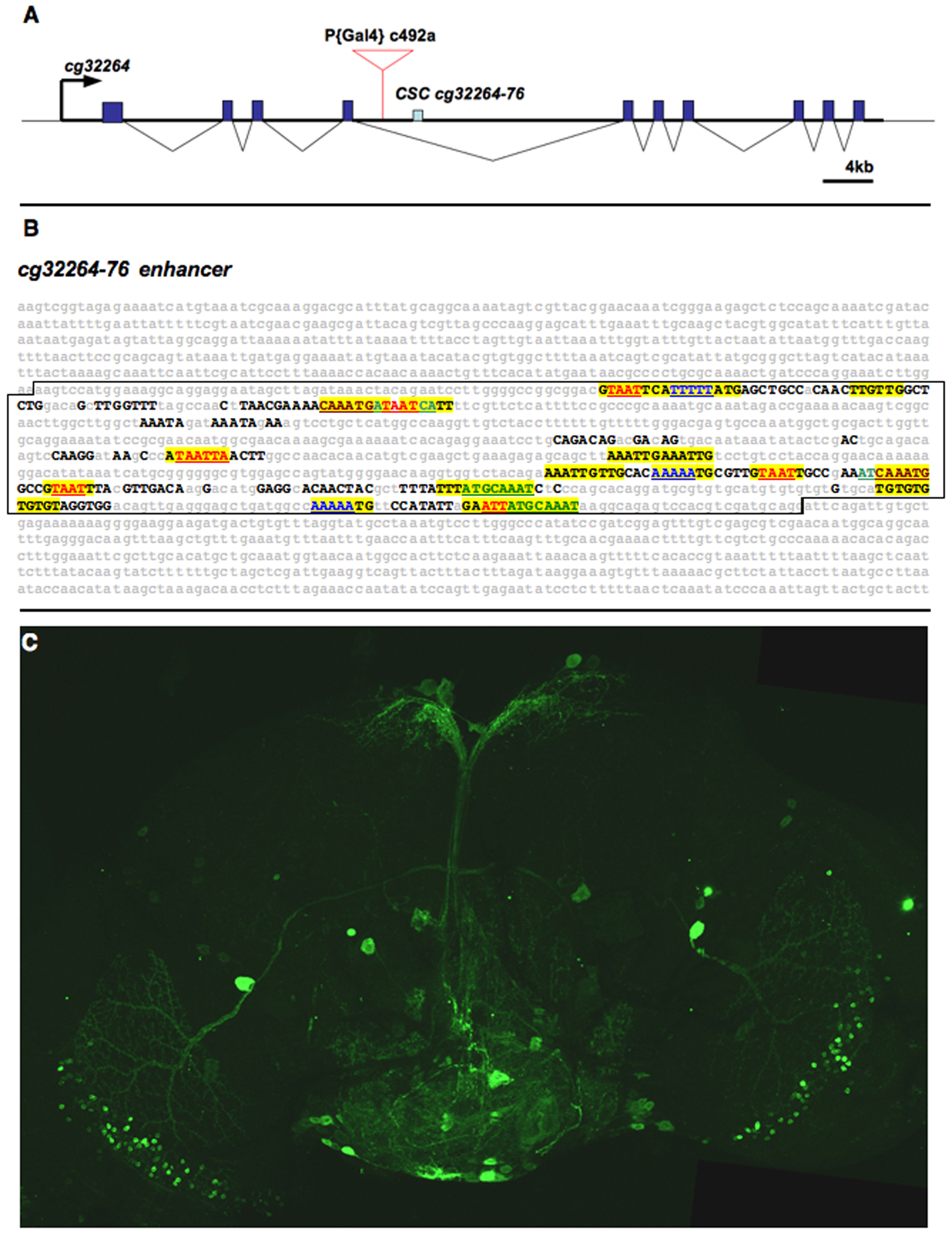

Supplement: Supplementary file 3 [file dvdy0241-0169-SD3.tif]

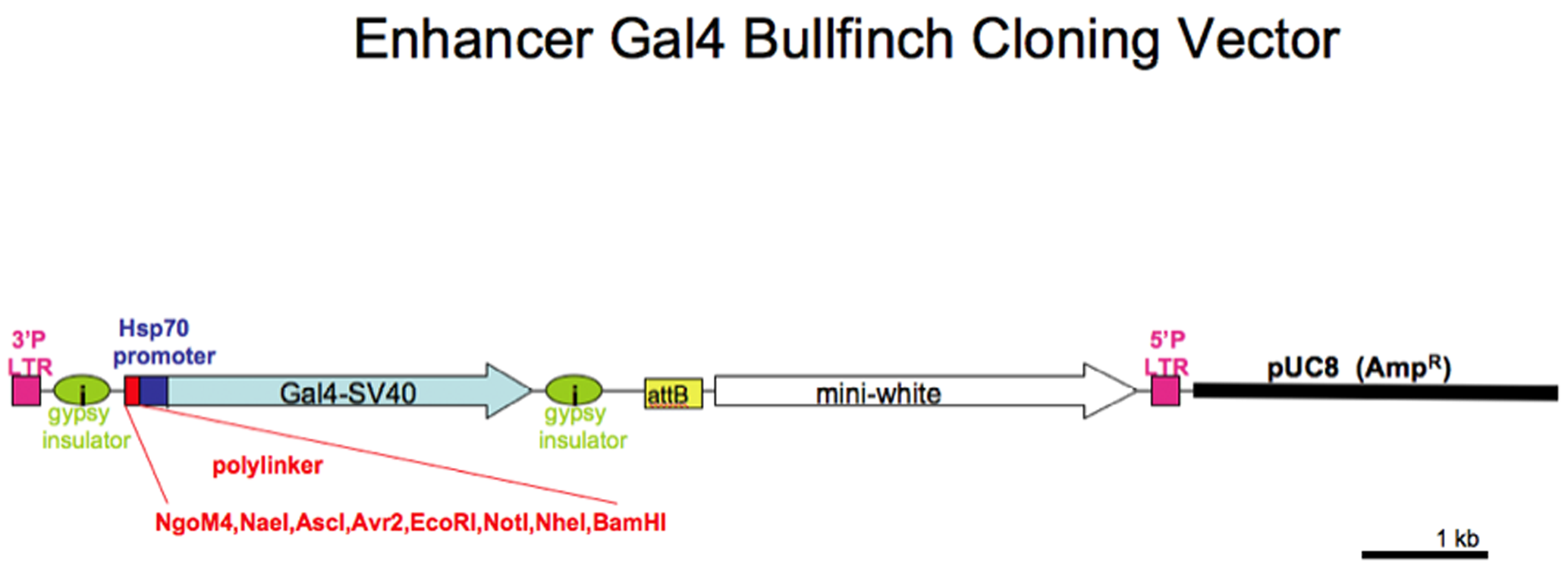

Supplement: Supplementary file 4 [file dvdy0241-0169-SD4.tif]
