# Supplementary material for: Use of a Drosophila Genome-Wide Conserved Sequence Database to Identify Functionally Related cis-Regulatory Enhancers
Source: Dev Dyn. 2011 Aug 30;241(1):169–89. doi: 10.1002/dvdy.22728 (PMC3243966; doi:10.1002/dvdy.22728)
Supplement: Supplementary file 5 [file dvdy0241-0169-SD5.pdf]

Table S1. Conserved sequence clusters: Location and cloning primers

CSC Name	location	Primers	Lines tested
cas-6	chr3R:1543220-1545461	5′end - AGATCTCAGTTACCAAAGCGGAAGCGTGCC<br>3′end - GCTAGCATCGGCAATCTTATTCGCC	>6
cg7229-5	chr3L:6778904-6779907	5′end - CTCGAGCTTAACGAGCGTTGAAAGCAACGCTTACCC<br>3′end - CGAAGGCATCATCATCAGCTGCAATCC	3
vvl-14	chr3L:6778896-6780034	5′end - GGCGCGCCTACTTAACTACTTAACGAGCG<br>3′end - GGATCCAAATAATCCTAGCATCGTCCTC	>2
nab-1	chr3L:4152555-4153566	5′end - GGCGCGCCAATACCTGACAATTGCCCTC<br>3′end - GGATCCTTTTTGAGTGCAAATATTGACACTCCTGGC	2
CG6559-28	chr3L:10300899-10302382	5′end - CTCGAGCTTTTCTCGATCGCAACGCG<br>3′end - CCGAACAACTAACGACAAGC	4
cas-8	chr3R:1546758-1549421	5′end - GCTAGCATGAAACCATCCCATCAGCGTG<br>3′end - AGATCTGCTGCTCAAGGACCGCCC	>6
tkr-15	chr2R:20988973-20990063	5′end - GGCGCGCCTAAAACGGTCCGTGGCCACC<br>3′end - AGATCTACCTAGATGATCGCATGTCACC	3
grh-15	chr2R:13716635-13718010	5′end - GGCGCGCCTGTGTCGGCTTCTTCAGCGG<br>3′end - GGATCCAGGATGGGTGGCTCCTTGCC	2
